# Modeling supply chain viability and adaptation against underload cascading failure during the COVID-19 pandemic

**DOI:** 10.1007/s11071-022-07741-8

**Published:** 2022-08-21

**Authors:** Hong Liu, Yunyan Han, Anding Zhu

**Affiliations:** 1 School of Computer and Information Engineering, Zhejiang Gongshang University, Hangzhou, 310018 People’s Republic of China; 2 School of Management and E-Business, Zhejiang Gongshang University, Hangzhou, 310018 People’s Republic of China; 3 Contemporary Business and Trade Research Center of Zhejiang Gongshang University, Hangzhou, 310018 China

**Keywords:** Supply chain viability, Supply chain adaptation, Underload cascading failure, Intertwined supply network, Complex adaptive system, COVID-19 economic disruption

## Abstract

Supply chain viability concerns the entire supply system rather than one company or one single chain to survive COVID-19 disruptions. Mobility restriction and overall demand decline lead to systematically cascading disruptions that are more severe and longer lasting than those caused by natural disasters and political conflicts. In the present study, the authors find that large companies and manufacturers with traditional advantages suffer greater losses than small ones, which is conceptualized as the “*Hub Paradox*” by empirically investigating one Warp Knitting Industrial Zone of China. An underload cascading failure model is employed to simulate supply chain viability under disruptions. Numerical simulations demonstrate that when the load decreases beyond a threshold, the viability will drop down critically. Besides, supply chain viability depends on two aspects: the adaptive capability of the manufacturers themselves and the adaptive capability of the connections of the supply network. The comparison study demonstrates that enhancing cooperative relations between hub and non-hub manufacturers will facilitate the entire supply network viability. The present study sheds light on viable supply chain management. Compared with conventionally linear or resilient supply chains, intertwined supply networks can leverage viability with higher adaptation of redistributing production capacities among manufacturers to re-establish overall scale advantages. Finally, the present study also suggests solving the “*Hub Paradox*” from the perspective of complex adaptive system.

**Supplementary Information:**

The online version contains supplementary material available at 10.1007/s11071-022-07741-8.

## Introduction

Supply chain viability (SCV) is defined as “the ability of a supply chain to maintain itself and survive in a changing environment through a redesign of structures and re-planning of performance with long-term impacts [1].” The term was coined in response to the supply chain disruption caused by the ongoing COVID-19 pandemic. As a concept comprehensively developed in natural ecosystems [2], viability has inspired scholars to think about supply chain resilience more dynamically and systematically than ever since 2020 [3–5]. The disruptions occasioned by the COVID-19 pandemic are unpredictable, bringing to light widespread lack of preparedness, and insufficient recovery capabilities, posing more challenges to risk management in the global supply chain than any previous natural catastrophes [6, 7] or political conflicts [8, 9].

A supply chain network (SCN) is a set of interconnected business entities, including external suppliers, manufacturing plants, distribution centers, demand zones, and transportation assets [10]. An SCN is a successive value-creation chain that collaborates to purchase materials, produce goods, and deliver goods to customers from the perspective of a single company. Meanwhile, many SCNs intertwine to form complex adaptive systems (CASs) [11, 12]. Complex network and graph theories have been applied to systematically analyze dynamic problems of vulnerability and robustness because SCNs are composed of numerous node entities and links of the material, information and capital flows [13–16]. A common dilemma of supply chain management is to obtain trade-offs between efficiency and robustness. A company prefers a simpler and more straightforward SC structure for better performance. On the other hand, robustness forces the company to arrange redundant inventory and suppliers to increase resilience [17]. From the point of view of the complex network theory, the redundant mechanism can enhance the capability of vulnerable node entities [13], which is particularly important in response to supply chain disruptions caused by natural disasters.

Japan is a central manufacturing hub of electronic components for the whole world. After the 2011 Great East Japan Earthquake and Tsunami, the global supply chains of semiconductor chips, dynamic random access memory (DRAM) chips, flash memory chips, standard logic controllers, and liquid crystal display (LCD) components were disrupted severely disrupted, immediately resulting in a 20% price increase (on average) of these components in the world market and subsequent shortages of critical components and operational shutdowns in American automaker plants (e.g., GM, Ford, and Chrysler) [7]. Japan suffers frequent earthquakes. In 2007, a magnitude 6.8 earthquake severely damaged a specialized piston ring manufacturer, which subsequently forced Toyota to shut down all twelve of its domestic assembly plants [6]. In complex network theory, an earthquake is represented as the removal of the hub from the network, resulting in cascading failures throughout the entire chain or network.

Political conflicts among countries also often pose threats to the robustness of global SCNs. The US-China trade war broke out recently, with each side in reciprocity increasing tariffs on imports from the other side. More severely, the US government banned Chinese telecommunication device manufacturers (e.g., Huawei) [9]. Banned from trading with US suppliers, Huawei had to replace US suppliers with China’s and other countries’ suppliers to reshape its supply chain rapidly [18]. Additionally, in 2001, the US government closed borders in response to large-scale terrorist acts, leading to the transportation disruptions of manufacturers operating the “Just-in-Time” (JIT) inventory discipline (e.g., Ford, Toyota, etc.) [8]. In complex network theory, a political conflict is represented as the removal of the connections between nodes or blocking the flow among nodes, resulting in cascading failures throughout the entire network.

In contrast, the COVID-19 pandemic systematically occasioned dynamic disruptions deep into every network entity and its links. In the early pandemic stage, the strategies of containment, isolation, lockdown, and quarantine reduced human mobility, subsequently leading to a precipitous drop in the consumption of goods and services [19]. For example, Nike officially stated on March 26, 2020, that stores would be closed in multiple countries worldwide, including the US, Canada, Western Europe, Australia and New Zealand [20]. On the supply side, Nike temporarily closed three factories of its second major supplier, Changshin Vietnam, which employs nearly 42,000 workers in Dong Nai province Vietnam, due to 177 infection cases detected in these factories [21]. COVID-19 has had systematically cascading effects on labor markets, healthcare sectors, gender and racial inequalities, as well as environmental consequences in almost every country worldwide [22]. As a modern economy is regarded as a complex network of interconnected parties [23, 24], Ivanov and Dolgui call on SC scholars to pay attention to the *viability* of SCNs in the post-pandemic era by emphasizing the specifically intertwined structure of SCNs [3].

To the best of our knowledge, the cascading failure in such an intertwined supply network (ISN) is different from those in supply chain disruptions caused by natural disasters and political conflicts. Drawing an analogy from the human body, a natural disaster may be compared to a disease that attacks one specific organ, while a political conflict sever organs one from the other. However, the disruption of COVID-19 may be compared to “bleeding to death” or “freezing to death.” Physiologically, sustained blood loss or hypothermia beyond a threshold will cause cascading injury which leads to shock.

Cascading failure is a critically systematic breakdown in complex networks [25, 26]. Many severe disruptions of real-world networks are often initialized by small errors, which accumulate to break down other adjacent parts, result in unbalanced loads, and successively bring the entire system into a crash. Cascading failures have been found in many real-world complex network systems, such as electric grids [27, 28], economic systems [29], and transportation systems [30]. There are two major node load attack modes in the cascading failure literature. One is overload attack. Every node in the network has its upper bound of capacity. When the node is overloaded, it will be destroyed permanently. Then, other parts will undertake the entire load, leading to their successive overloads [31, 32]. The other is underload attack. Every node in the network also has its lower bound of capacity. When the flow is too low to maintain the regular operation of the node, it will also be closed, sometimes leading to higher operation costs of other parts. Underload cascading failures often take place in SCNs [15, 16].

However, COVID-19 poses a new situation to the systematic viability of SCN disruptions. Ivanov suggested four adaptation strategies (i.e., intertwining, scalability, substitution, and repurposing) to maintain SCV when facing the COVID-19 pandemic [33]. Altan and Karasu proposed a deep learning technique to recognize COVID-19 risks for optimizing adaptation strategies [34]. Karasu et al. proposed a novel forecasting approach for predicting the chaotic crude oil demand caused by COVID-19 [35]. In addition, inventory renewal optimization approaches [36], rapid feedback strategies [37], supply chain finance [38], system identification approaches [39] were proposed to adapt to the demand fluctuations and mitigate supply chain disruptions brought by COVID-19. On the other hand, Ivanov and Dolgui [3] and Pimenta et al. [40] suggested mapping capabilities of supply chain resilience in a systemic framework. Butt conducted the multiple case analysis presenting the comprehensive cooperation between distributors [41]. Similarly, the present study found the adaptive capabilities of the supply chain by investigating the dynamically material flows of the Warp Knitting Industrial Zone (WKIZ) in Northeast Zhejiang Province, China, after the COVID-19 pandemic. Then, we adapt the underload cascading failure model to reveal the viability mechanism of adaptive load redistributions among manufacturers.

The rest of the paper is arranged as follows: The mechanism of the intertwined supply network and one case study are presented in Sect. 2. Then, a viability model against underload cascading failures is proposed in Sect. 3. Numerical simulations and a comparative study are conducted in Sect. 4. Finally, conclusions and suggestions are presented to improve supply chain viability in Sect. 5.

## The intertwined supply network

In this section, the concept and characteristics of an intertwined supply network are demonstrated, followed by a case study on one warp knitting supply network in China. The empirical analysis of the warp knitting supply network inspires the viability model in the subsequent section.

### The intertwined structure

**Fig. 1:**
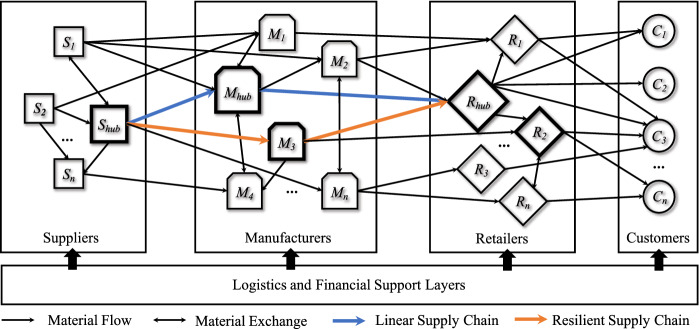
A supply chain network with an intertwined structure

As shown in Fig. 1, a four-echelon SCN comprises suppliers, manufacturers, retailers, and customers. Complex network theory regards entities as nodes and their relations as edges. Generally, SCNs are directed networks. There are many types of flows among entities, e.g., material flow, information flow, capital flow, etc. [15]. Only material flow is considered in the present study, including flows of raw materials, partly finished goods, finished goods, packages, etc.

Conventional SCN models primarily only consider nodes within the same layer as homogeneous actors for simplicity. The nodes are heterogeneous in scale and connection even in the same layer. For example, the hubs in the “Suppliers,” “Manufacturers,” and “Retailers” layers are labeled $S_{hub}$, $M_{hub}$, and $R_{hub}$, respectively. Furthermore, smaller quasi-hubs in the layers are labeled $M_3$ in the “Manufacturers” layer and $R_2$ in the “Retailers” layer.

In a real-world SCN, there are connections among nodes across two adjacent layers and many connections among nodes within the same layers. For example, although $S_1$, $S_2$, ..., $S_n$ are all suppliers, they also have cooperative relations. Imagine one scenario: the hub supplier $S_{hub}$ receives an order but cannot schedule it. $S_{hub}$ has a good cooperative relation with supplier $S_2$. Then, $S_{hub}$ outsources the order to $S_2$. Therefore, we will find a material flow from $S_2$ to $S_{hub}$. Imagine another scenario: the small supplier $S_1$ receives a larger order that exceeds its production capacity. $S_1$ outsources the order to $S_{hub}$ because the latter has a good reputation and quality guarantee. Therefore, we will find a material flow from $S_{hub}$ to $S_1$. Of course, $S_{hub}$ may also outsource orders to $S_1$. Therefore, they have reciprocal relations, forming bidirectional material flows. The third scenario is that two non-hub nodes cooperate. For instance, supplier $S_n$ outsources orders to supplier $S_2$, forming a material flow from $S_2$ to $S_n$. Cooperation and synergy among nodes within the same layer are common and widely exist. It can be regarded as the exchange of production capacities to improve the overall performance. Most of the time, these exchanges are autonomous to search the routes of minimizing time, production, and transaction costs. In this sense, the complex cooperation system within the same layer is also a complex adaptive system (CAS). This type of cooperation is more complex and significant in the “Manufacturers” layer because the number of manufacturers is much larger than that of suppliers.

Extant studies emphasize the material flows passing from upstream layers to downstream layers [10, 13, 15]. For a simply linear supply chain (marked with blue arrows in Fig. 1), the material flows only pass through one node in one layer (e.g., $S_{hub}\rightarrow M_{hub}\rightarrow R_{hub}$). Many large international companies, such as Nike and IKEA, prefer keeping their supply chains short and straightforward to minimize procurement costs. Therefore, they prefer to establish long-term and stable relations with hubs. On the other hand, potential supply chain interruptions, such as earthquakes, motivate companies to establish resilient supply chains by redundant links (e.g., $S_{hub}\rightarrow M_{3}\rightarrow R_{hub}$ marked with orange arrows in Fig. 1).

Ivanov and Dolgui propose that the concept of an “Intertwined Supply Chain” (ISN) which defined as “an entirety of interconnected supply chains which, in their integrity secure the provision of society and markets with goods and services.” In addition, they write, “an ISN is a complex supply network with dynamically changing structures, roles and behaviors of the firms involved [3].” ISNs try to capture the nature of complex and dynamic connectedness in real-world supply networks. For instance, $S_1$ supplies $M_1$, $M_2$, and $M_{hub}$. Meanwhile, $R_{hub}$ has three suppliers: $M_2$, $M_3$, and $M_{hub}$. In addition, $R_{hub}$ sells goods to three customers: $C_1$, $C_2$, and $C_3$.

Facing the COVID-19 disruptions, even with the redundant supplier of $M_3$, retailer $R_{hub}$ still has breakdown risk according to the local lockdowns of $M_{hub}$ and $M_3$. For $R_{hub}$, a more comprehensive cooperation network within the “Manufacturers” layer will help it survive even losing the pre-designed resilience. The other scenario is when the sales of $R_{hub}$ (e.g., Nike, Adidas, etc.) decline rapidly and continuously, its suppliers of $M_{hub}$ and $M_3$ are unable to obtain sufficient orders to cover their bottom lines. Then, they may choose to shut down, leading to supply chain interruptions of $R_{hub}$, and even $R_2$.

Hub manufacturers always have scale effects of having a higher chance of becoming the major suppliers for the downstream hub retailers. Generally, hub manufacturers have many advantages, such as advanced machinery and equipment, skilled labor, higher management, and scheduling capability, resulting in higher quality guarantees, better delivery management, and lower costs and prices. Similarly, hub retailers also have a scale effect because they collect a great demand into integrated orders. Therefore, hub retailers prefer to associate with hub manufacturers. However, when hub retailers fail to gather consumer demand due to the containment strategies during the COVID-19 pandemic, hub manufacturers will lose their stable orders and be forced to shut down.

We call this phenomenon the “*Hub Paradox*”. Intuitively, the small manufacturers are supposed to be more vulnerable and less sustainable than strong hub manufacturers during disruptions. However, the reality is that the strong hub manufacturers may be out first. It is not reasonable and sensible. Why can hub manufacturers produce higher-quality goods at lower prices but not survive? Why can’t there be channels for capacity exchange between hub manufacturers and small ones? If so, hub manufacturers can overcome the difficulties. Meanwhile, small manufacturers and retailers can also benefit. Therefore, the intertwined supply network is expected to facilitate viability and adaptation more than conventionally linear and resilient supply chains.

### Case study

One Warp Knitting Industrial Zone (WKIZ) located in Northeast Zhejiang Province, China, was investigated after the outbreak of COVID-19 in 2020. Warp knitting is a crucial kind of textile technology. WKIZ is one of China’s major supplier clusters of fabrics for many international sportswear brands (e.g., Uniqlo, Nike, Zara, Adidas, Decathlon, Fila, etc.). In WKIZ, there are three categories of plants: (1) chemical fiber, yarn, and innovative material producing plants (belonging to suppliers); (2) weaving plants (manufacturers of the first process), dyeing plants (manufacturers of the second process), and finishing plants (manufacturers of finished fabrics), which belong to manufacturers; and (3) trading companies and service companies (belonging to intermediaries). After four echelons of fiber production, weaving, dyeing, and finishing, the finished fabrics will further be used to produce clothes, household textiles, and industrial applications (e.g., advertising printing cloth and highway road bases) in the zone or out of the zone.

According to Yearbook 2020 authorized by WKIZ, the outbreak of COVID-19 resulted in sudden drops of $-32.2\%$ and $-24.3\%$ of WKIZ’s output during February and March, respectively. In the remaining ten months of 2020, the decline rate remained stable at approximately $-10\%$ because the Chinese government adopted efficient containment measures. Table 1 lists the average revenue growth rates of 249 companies located in WKIZ (the primary dataset is available in the supplemental material section). Among 249 companies, 207 are manufacturing plants, while 42 are trading intermediaries. Only 150 plants specialize in one process (e.g., fiber production, weaving, dyeing, finishing, and clothing). The other 57 plants integrate different processes to increase flexibility (e.g., fiber production and weaving, finishing, etc.). Trading companies also have specialities (e.g., fiber, clothes, etc.). When they obtain orders from customers, they find suitable plants to complete the production. Trading companies take advantage of extensive connections with manufacturers to provide customers with higher service quality. We cannot obtain the WKIZ’s supply network structure because it involves business secrets and is constantly changing. However, we realize that the WKIZ’s supply system is a complex ecosystem with an intertwined network structure.

**Table 1:** The average revenue growth of 249 companies in the WKIZ by category

Categories	Number of	Total revenue	Average revenue
	companies	(normalized)	growth rate %
Fiber supplier	20	2.3984	−3.76
Weaving	30	1.3916	0.75
Dyeing	9	0.6169	22.52
Finishing	81	2.3127	−5.37
Clothing	10	0.2527	−28.29
Fiber supplier & weaving	3	0.0641	−25.95
Fiber supplier & finishing	10	0.3161	−9.88
Fiber supplier & weaving & Finishing	1	0.0086	−40.93
Weaving & dyeing	7	0.7285	−15.78
Weaving & finishing	26	0.6659	−11.44
Weaving & dyeing & finishing	1	0.0854	−17.20
Finishing & clothing	6	0.0820	−23.52
Finishing & dyeing	3	0.0338	−7.08
Trading (fiber)	9	1.3294	−1.97
Trading (fiber & finished goods)	1	0.0232	4.40
Trading (finished goods)	30	1.6219	−5.90
Trading (finished goods & clothes)	1	0.0246	−21.20
Trading (clothes)	1	0.0791	−8.30

**Fig. 2:**
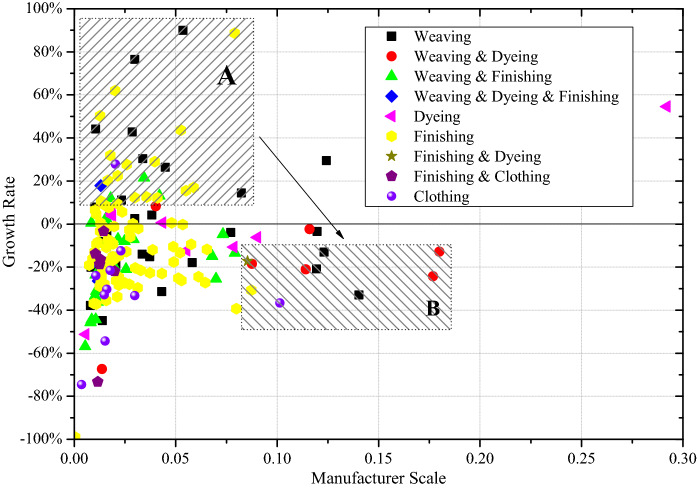
The growth rates of the manufacturer category in WKIZ (2020)

As listed in Table 1, the clothing plants reduced production the most (−28.29% on average) due to the sharp decline in orders from major international brands. We now know that consumers switched their consumption habits to online shopping, leading to the sales increase in online small brands. In addition, the finishing plants and fiber suppliers were reduced too. Instead, dyeing plants increased because of the capacity restrictions for the environmental protection. The weaving plants did not present a significant decline in production on average. However, COVID-19 caused further losses to manufacturers. The growth rates of several large weaving and weaving & dyeing plants dropped to negative output (shown as rectangle B in Fig. 2). These hub manufacturers had higher production capacities, but lost batch orders, which is conceptualized as the “*Hub Paradox*” above. On the other hand, many small plants produced small orders for e-commerce sellers and achieved favorable growth rates (shown as rectangle A in Fig. 2). As an adaptive system, the small plants are motivated to adopt large plants’ production capacities, achieving a win-win situation and improving the overall performance of the supply system.

## The viability model against underload cascading failure

A cascading failure is a successive process starting from initial breakdowns of some nodes and spreading to a larger number of other nodes. Mitigation and recovery strategies are found in the literature. Wang demonstrated the strategy of adjusting the overload edges to improve the robustness level against cascading failures in scale-free networks [25]. Zhang et al. proposed employing the link prediction approach in redistributing load dynamically to mitigate cascading failure disruptions [42]. Zhong et al. evaluated the network endurance against overload cascading failures and found that initial disturbance intensity and cascade intensity are the critical factors impacting endurance [43]. Yang et al. found a discontinuous phase transition against load fluctuations [16].

This study will simulate the autonomous mechanism to achieve higher adaptation in a viable supply network system against underload cascading failures. The “Manufacturer” layer is supposed to have an intertwined network structure that can be modeled as a bidirectional and weighted graph, $G_{ISN}=(V_N, E_M, W_M)$. Therein, $V_N=\{v_1, v_2, ..., v_N\}$ denotes the set of nodes (i.e., manufacturers); $E_M=\{e_1, e_2, ..., e_M\}$ denotes the set of edges (i.e., material flows between manufacturers); and $\{W_M=w_1, w_2,..., w_M\}$ denotes the set of weights on edges (i.e., the intensities of material flows). *N* and *M* represent the number of nodes and edges, respectively. We use material flows to represent outsourcing relations among manufacturers. In addition, $e_{ij}$ denotes the directed edge of material flowing from node $v_i$ to node $v_j$. The physical meaning is that manufacturer $v_j$ outsources an order to manufacturer $v_i$. Obviously, $e_{ij}\in E_M$. In addition, $d_i$ denotes the degree of node $v_i$, $A_i$ denotes the set of adjacent nodes of $v_i$, and the relation intensity between nodes $v_i$ and $v_j$ is supposed to be $w_{ij}=(d_{i}d_{j})^\lambda$ ($\lambda$ is an adjustment parameter) [44].

### Initial load

The node’s initial load is set according to its importance. The literature on cascading failures provides two categories of initial load setting, i.e., degree-based approaches [45] and betweenness-based approaches [46, 47]. A manufacturer depends on other close connections in an ISN scenario. Therefore, one node’s initial load is supposed to be highly related to the loads of other neighboring nodes. From the side of node $v_i$, $I_{i}$ denotes the degree of edges flowing in node $v_i$, while $O_{i}$ denotes the degree of edges flowing out of node $v_i$. Then, node $v_i$’s initial load ($L_i^0$) can be set by Formula 1 [48].

1 $$\begin{aligned} L_{i}^0=[d_{i}\sum \nolimits _{j\in A_{i}}d_{j}]^\alpha , i, j=1, 2, ..., N, \end{aligned}$$

where $d_{i}=I_{i}+O_{i}$ is the total degree of node $v_i$; $A_{i}$ represents the set of nodes adjoined to node $v_i$; $d_{j}$ represents the degree of node $v_i$’s adjacent node $v_j$; and $\alpha ~(0\le \alpha \le 1)$ is the *initial intensity factor* to initialize the intensity of node $v_i$’s load.

### Bounds of load

In the real-world SCNs, the manufacturer has its upper bound and lower bound of production capacity. When the orders exceed the upper bound, the manufacturer cannot fulfill the orders by itself. In the present study, node $v_i$’s upper bound ($CU_i$) is supposed to be proportional to its initial load $L_{i}^0$ [49, 50]:

2 $$\begin{aligned} CU_{i}=\beta L_{i}^0, i=1, 2, ..., N, \end{aligned}$$

**Fig. 3:**
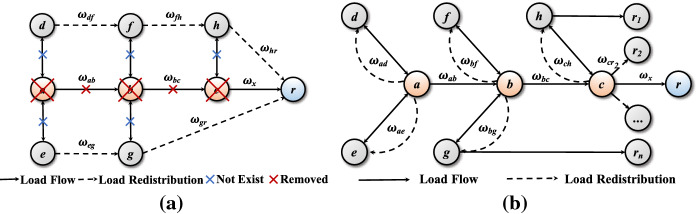
Load redistribution after the decline of large-scale orders from international brand retailers. **a** The situation of hub removals. **b** The situation of autonomous redistribution of load

where $\beta ~(\beta \ge 1)$ is defined as the *upper bound factor* of node $v_i$’s production capacity. The larger the value of $\beta$ is, the larger the production scale of node $v_i$.

Meanwhile, manufacturers are also restricted by fixed costs. A manufacturer will operate at a loss if the quantity of orders is too low to cover the fixed costs. When the manufacturer predicts or suffers from insufficient orders, it may choose to shut down. Therefore, we also suppose that node $v_i$’s lower bound ($CL_i$) is proportional to its initial load $L_{i}^0$ [44]:

3 $$\begin{aligned} CL_{i}=\gamma L_{i}^0, i=1, 2, ..., N, \end{aligned}$$

where $\gamma ~(0\le \gamma \le 1)$ is defined as the *lower bound factor* of node $v_i$’s production capacity. The smaller the value of $\gamma$ is, the stronger the endurance of node $v_i$.

### Autonomous redistribution of load

Hubs were first threaten and the loads declined rapidly after the pandemic. Hub manufacturers may have to be closed if the loads decline to below their bottom line (i.e., $L_{i}<CL_{i}$). The invalidity of the hubs will negatively impact the entire network.

As shown in Fig. 3a, hubs *a*, *b*, and *c* make up a tightly coupled supply chain for an external hub retailer *r*. Typically, it is enough for hubs *a*, *b*, and *c* to survive and make significant profits as long as they cooperate tightly to serve hub retailer *r*. If they exclude non-hub nodes from their cooperative lists, they will have no links with non-hub nodes (marked with blue crosses). They may consider shutting down when the epidemic comes due to the sudden and successive decrease in orders from hub retailers ($w_x$) (marked with red crosses). Subsequently, retailer *r* needs to establish other routines to fulfill orders. The updated routines of $w_{df}\rightarrow w_{fh}\rightarrow w_{hr}$ and $w_{eg}\rightarrow w_{gr}$ are both alternatives (marked with dashed arrows). The loads initially flowing through hubs *a*, *b*, and *c* will be redistributed to nodes *d*, *f*, *h* or *e*, *g*.

However, as mentioned above, hubs have intensive and broad cooperation with others in an intertwined supply network. Hub manufacturers can perform adaptively by undertaking orders from other non-hub nodes to achieve win-win cooperation with their competitive advantages.

As a comparison with Fig. 3a, b demonstrates another scenario of load redistribution. When COVID-19 breaks out, the external retailer *r* rapidly reduces its orders, leading to a load decrease on hub *c*. Subsequently, hubs *b* and *a* suffer the load decreases via the edges of $w_{bc}$ and $w_{ab}$, respectively. If hubs *a*, *b*, and *c* have already established cooperative relations with other non-hub nodes (represented by bidirectional arrows), they have more opportunities to take outsourcing orders from adjacent nodes to maintain their economic production capacity. For instance, hub *a* provides manufacturing services for nodes *d* and *e*; hub *b* provides manufacturing services for nodes *f* and *g*; hub *c* provides manufacturing services for node *h* and directly fulfills orders from non-hub retailer $r_2$ and others (marked with dashed arrows). Non-hub nodes *h* and *g* take orders from many non-hub retailers $r_1$ and $r_n$. In this scenario, the load redistributions of $w_{ad}$, $w_{ae}$, $w_{bf}$, $w_{bg}$, $w_{ch}$, and $w_{cr_2}$ provide hubs *a*, *b*, and *c* extra orders to cover the loss from hub retailer *r*. Hubs will get over the disruptions, and the entire supply network will survive at a low-level production capacity.

In the present study, we adapt the load redistribution to simulate the adaptive mechanism as follows [15, 16]:

1. Given a weighted directed graph *G* with *N* nodes and *M* edges, set the basic parameters of the *initial intensity factor* ($\alpha$), *upper bound factor* ($\beta$), and *low bound factor* ($\gamma$). Then, calculate the degree ($d_i$), initial load ($L^0_i$), upper bound ($CU_i$), and lower bound ($CL_i$) for each node $v_i$ according to the Formulas 1, 2, and 3.
2. Sort all nodes in descending order of degree and choose the top *k* connected nodes into the hub set *K*.
3. Set the *load decrease ratio* ($\rho$) and calculate the load decreases of *k* hubs at time *t* with $\varDelta L_{k\in K}(t)=\rho L^0_k$. Then, update hubs’ loads with $L_{k\in K}(t)=(1-\rho )L^0_k$.
4. Set the *capacity adjustment parameter* (*CP*) and adjust the upper bound of each hub with $CU_{k\in K}(t)=CP\cdot CU_{k\in K}$ to reduce its maintenance cost.
5. Choose one hub $v_k$ from *K*.
6. Put all the adjacent upstream nodes of $v_k$ into set: $A^{\prime }_k=\{v_j|v_j\in A_k \wedge w_{kj}\ne 0\}$.
7. Choose one node $v_j$ from $A^{\prime }_k$. We suppose that non-hub nodes with higher redundant capacity will be chosen with higher priority. The redundant capacity of a node can be measured by the difference between upper bound of production capacity and the current load [51]. Calculate the redundant capacity of node $v_j$ with $R_j(t)=CU_j(t)-L_j(t)$. Then, redistribute $v_j$’s load to hub *k* with $\varDelta L_{jk}=min(\varDelta L_k, R_k(t))$.
8. Update node $v_j$’s weight and load with formulas of $w_{jk}(t+1)=w_{jk}(t)\frac{L_j(t)+\varDelta L_{jk}}{L_k(t)}$ and $L_j(t+1)=L_j(t)+\varDelta L_{jk}$, respectively. Update node $v_k$’s load with formulas of $\varDelta L_k=\varDelta L_k-w_{jk}(t)\frac{\varDelta L_{jk}}{L_k(t)}$ and $L_k(t+1)=L_k(t)-\varDelta L_k$.
9. At time $t+1$, if $L_k(t+1)<CU_k(t+1)$, update $A^{\prime }_k-\{v_j\}$. Then, if $A^{\prime }_k\ne \emptyset$, jump to step 7. If $A^{\prime }_k=\emptyset$, update $K=K-\{v_k\}$ and jump to step 5. If $L_k(t+1)\le CU_k(t+1)$, update $K=K-\{v_k\}$ and jump to step 5. If $L_j(t+1)<CL_j$, hub *k* will fail.
10. Finally, calculate the viability index (see Sect. 3.4).

### The viability index

There is no viability index in the literature. With the progression of COVID-19, hub loads often vary from initial loads or lower boundaries of load capacities. Therefore, one node has three states: normal state, underload state, and failure state. When an increasing number of hubs fail before smaller nodes, the “*Hub Paradox*” occurs. A simple viability index *V* is defined as Formula 4, by adapting the failure rate from Ref. [52]:

4 $$\begin{aligned} V=1-\frac{\text {the~number~of~failure~hubs}}{\text {the~total~number~of~hubs}}. \end{aligned}$$

The larger the value of *V* is, the higher the viability and endurance of the supply network system.

## Numerical simulations

Extant studies have demonstrated that supply chain networks have scale-free characteristics [53, 54]. In the present study, we compared the viability of the supply network system against underload cascading failures on Barabási-Albert (BA) model [55] and Erdös-Rényi (ER) model [56]. According to Ref. [44], $\lambda$ is set to 0.5. The artificial networks are generated with 500 nodes and 2, 000 directed edges. Each simulation is conducted 20 times, and the results are averaged.

### The impact of the *initial intensity factor* on the viability

**Fig. 4:**
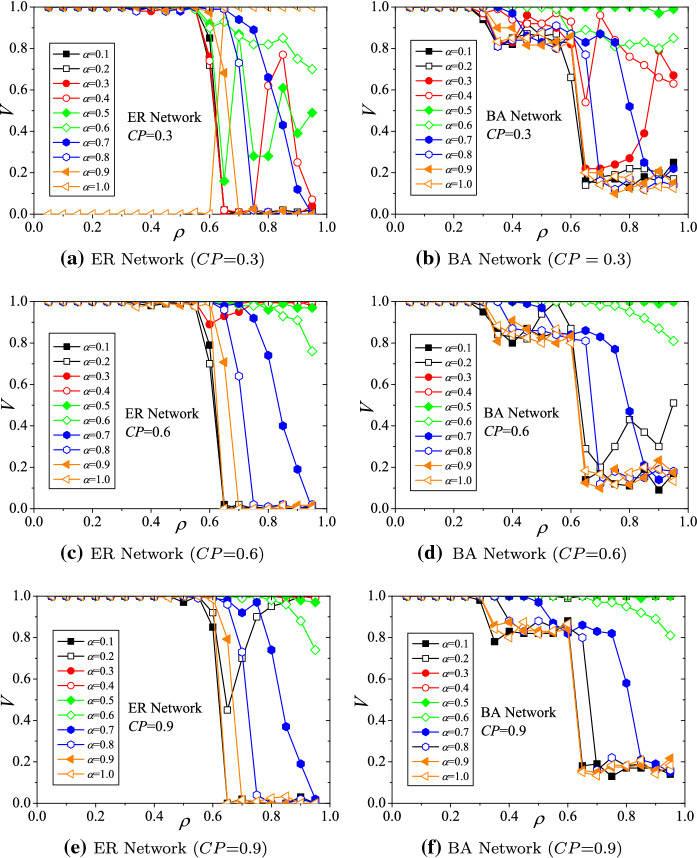
Hub viability against underload cascading failures with respect to initial intensity factor ($\alpha$)

The parameter settings for these simulations are $\beta =1.5$, $\gamma =0.4$, and $CP=0.3, 0.6$, and 0.9. The simulation results of the hub viability against underload cascading failures on ER and BA structured supply networks with respect to *initial intensity factor* ($\alpha$) are demonstrated in Fig. 4. Generally, with different $\alpha$s, the viability of hubs declines with increasing *load decreasing ratio* ($\rho$) (shown in Fig. 4a–f). Evaluations of viability present the phenomenon of phase transition. For ER networks (Fig. 4a, c, e), the viability indices (*V*s) drop down rapidly when $\rho >0.6$. A similar critical phenomenon also exists in BA networks. As shown in Fig. 4b, d, f), when $\rho$s fall into the range of (0.3, 0.6), the viability indices drop to a platform near 0.8. When $\rho >0.6$, the viability indices drop down critically.

On the other hand, raising the *capacity adjustment parameter* will improve the situation of hub viability. For instance, for ER networks, when $\alpha =0.5$, $\rho =0.75$, and $CP=0.3$, the viability index $V= 0.28$ (demonstrated in Fig. 4c). When *CP* is adjusted to 0.6, *V* increases nearly to 1.0 (demonstrated in Fig. 4a). Similar situations exist in BA networks. When $\alpha =0.3$, $\rho =0.65$, and $CP=0.3$, the viability index $V=0.22$ (demonstrated in Fig. 4d). When *CP* is adjusted to 0.6, *V* increases nearly to 1.0 (demonstrated in Fig. 4b). Therefore, the results implicate that the stronger the adjustment capabilities of hubs are, the higher the viability of the hubs, because the loads redistributed from non-hubs can cover the losses of underload cascading failures on hubs.

### The impact of the *capacity adjustment parameter* on the viability

**Fig. 5:**
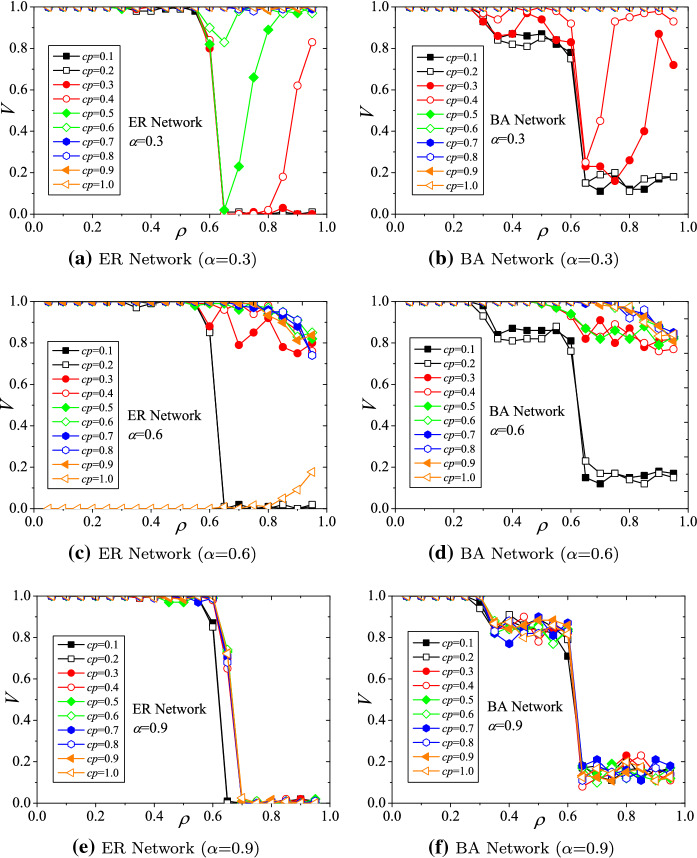
Hub viability against underload cascading failures with respect to capacity adjustment parameter (*CP*)

The parameter settings for these simulations are $\beta =1.5$, $\gamma =0.4$, and $\alpha =0.3, 0.6$, and 0.9. The simulation results of the hub viability against underload cascading failures on ER and BA structured supply networks with respect to *capacity adjustment parameter* (*CP*) are demonstrated in Fig. 5. Generally, with different *CP*s, the viability of hubs declines with increasing *load decreasing ratio* ($\rho$) (shown in Fig. 5a–f). Evaluations of viability also present the phenomenon of phase transition. For ER networks (Fig. 5a, c, e), the viability indices (*V*s) drop down rapidly when $\rho >0.5$. Similarly, for BA networks (Fig. 5b, d, e), the viability indices drop slightly when $\rho$s fall into the range of (0.3, 0.6). When $\rho >0.6$, the viability indices drop down critically.

On the other hand, raising the *initial intensity factor* will improve hub viability. For instance, for ER networks, when $\alpha =0.3$, $\rho =0.65$, and $CP=0.5$, the viability index $V\approx 0.0$ (demonstrated in Fig. 5a). When $\alpha$ is adjusted to 0.6, *V* improves closely to 1.0 (demonstrated in Fig. 5c). Similar situations exist in BA networks. When $\alpha =0.3$, $\rho =0.65$, and $CP=0.3$, the viability index $V\approx 0.23$ (demonstrated in Fig. 5b). When $\alpha$ is adjusted to 0.6, *V* improves to about 0.82 (demonstrated in Fig. 5d). Therefore, the results implicate that the larger the value of $\alpha$ (i.e., the larger the initial intensity of load) is, the larger the redundant capability of non-hubs, so that the hubs can adopt more loads from connected non-hubs to cover the losses of underload cascading failures.

### The impact of the *upper bound factor* on the viability

**Fig. 6:**
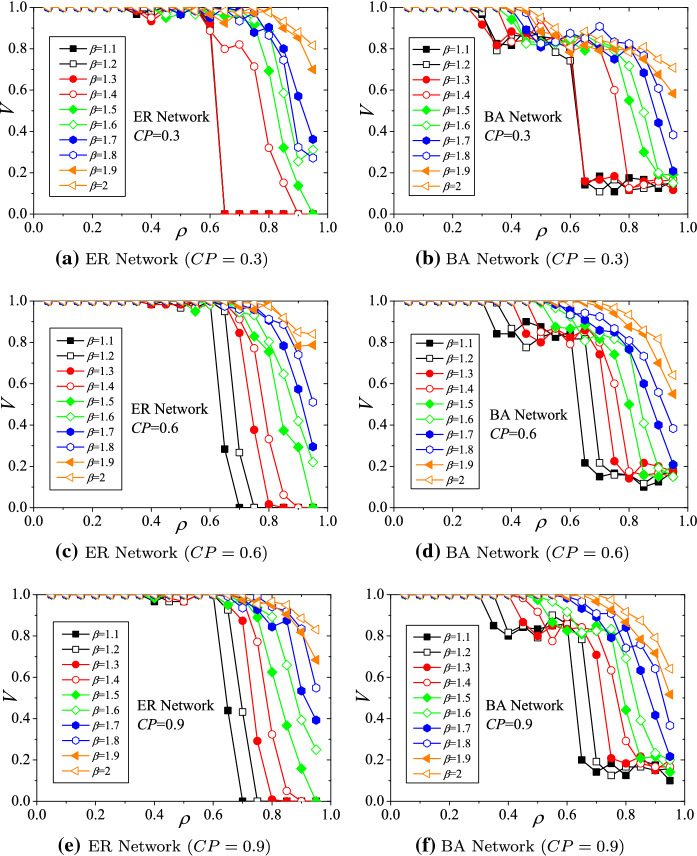
Hub viability against underload cascading failures with respect to upper bound factor $\beta$

The parameter settings for these simulations are $\alpha =0.7$, $\gamma =0.4$, and $CP=0.3, 0.6$, and 0.9. The simulation results of the hub viability against underload cascading failures on ER and BA structured supply networks with respect to *upper bound factor* ($\beta$) are demonstrated in Fig. 6. Generally, with different $\beta$s, the viability of hubs declines with increasing *load decreasing ratio* ($\rho$) (shown in Fig. 6a–f). Evaluations of viability also present the phenomenon of phase transition. For ER networks (Fig. 6a, c, e), the viability indices (*V*s) drop down rapidly when $\rho >0.6$. For BA networks (Fig. 6b, d, f), the viability indices decline to a platform near 0.8 when $0.3<\rho <0.6$. Then, the viability indices drop down critically when $\rho >0.6$. For the same *CP* and $\rho$, *V*s are positively related to $\beta$s, which implies that the larger the value of $\beta$ (i.e., the larger the upper bound of the production capacity) is, the more viable the hub manufacturers are to deal with the disruptions.

In addition, for the same $\beta$, the hub viability will be leveraged by increasing the value of *CP*. For example, for ER networks, $V=0.8$ when $\beta =1.6$, $\rho =0.8$, and $CP=0.6$ (shown in Fig. 6c). When *CP* increases to 0.9, *V* increases to 0.89 (shown in Fig. 6e). Similarly, for BA networks, $V=0.86$ when $\beta =1.7$, $\rho =0.7$, and $CP=0.6$ (shown in Fig. 6d). When *CP* increases to 0.9, *V* increases to 0.89. Therefore, the results indicate that hub manufacturers can leverage viability by adjusting their production capabilities and enhancing the relations with non-hub manufacturers.

### The impact of the lower bound factor on the viability

**Fig. 7:**
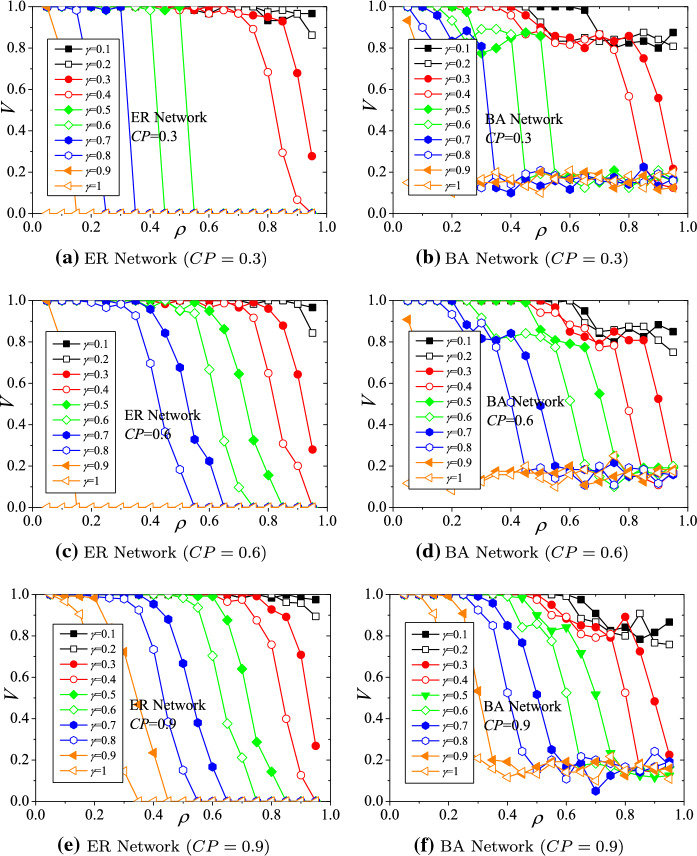
Hub viability against underload cascading failures with respect to *lower bound factor* ($\gamma$)

The parameter settings for these simulations are $\alpha =0.7$, $\beta =1.5$, and $CP=0.3, 0.6$, and 0.9. The simulation results of the hub viability against underload cascading failures on ER and BA structured supply networks with respect to the *lower bound factor* ($\gamma$) are demonstrated in Fig. 7. Generally, with different $\gamma$s, the viability of hubs declines with increasing *load decreasing ratio* ($\rho$) (shown in Fig. 7(a–f). For the same *CP* and $\rho$, *V*s are negatively related to $\gamma$s, which implies that the smaller the value of $\gamma$ (i.e., the smaller the lower bound of the production capacity) is, the lower the cost of maintaining normal production so that the more viable the hub manufacturers are to deal with the disruptions.

In addition, for the same $\gamma$, the hub viability will be leveraged by increasing the value of *CP*. For example, for ER networks, $V=0.649$ when $\gamma =0.4$, $\rho =0.8$, and $CP=0.6$ (shown in Fig. 7c). When *CP* increases to 0.9, *V* increases to 0.758 (shown in Fig. 7e). Similarly, for BA networks, $V=0.783$ when $\gamma =0.4$, $\rho =0.75$, and $CP=0.6$ (shown in Fig. 7d). When *CP* increases to 0.9, *V* increases to 0.808. On the other hand, the actually fixed costs cannot be too high (i.e., the value of $\gamma$ cannot be too large), or the viability will be very low. For ER networks, when $\gamma >0.9$, *V*s tend to 0 (shown in Fig. 7a, c, e). Similarly, for BA networks, when $\gamma >0.9$, *V*s tend to 0.1 (shown in Fig. 7b, d, f). Therefore, the results indicate that hub manufacturers with higher fixed costs will be less likely to survive the disruptions caused by COVID-19, even if they enhance the cooperative relations with non-hub manufacturers or adjust their production capabilities.

### The comparison of the viability

**Fig. 8:**
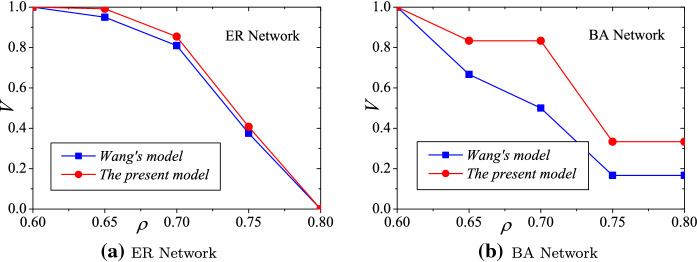
The comparison between two models

An additional performance verification compares two models: the underload failure cascading model proposed by Wang in Ref. [15] and the present model. The parameter settings are $\alpha =0.7$, $\beta =1.3$, $\gamma =0.4$, and $CP=0.7$. As illustrated in Fig. 8, the present model achieves better hub viabilities than Wang’s model in both ER and BA networks. The present model focuses on enhancing the cooperative relations among hub and non-hub manufacturers rather than redistributing loads to manufacturers along the supply chain in Wang’s model [15], when the entire supply network suffers the rapid decline of orders. According to the load redistribution mechanism of the present model, the hub manufacturers can leverage viability by taking orders from non-hub manufacturers and adjusting their production capabilities, e.g., shutting down some assembly lines. However, when the demand declines to an extreme level, for example, $\rho =0.8$ in Fig. 8, the hub viabilities of the two models will both be very low. Therefore, the comparison results imply that comprehensive cooperation within the entire supply network will facilitate the performances of both hub and non-hub manufacturers when they face the overall disruptions caused by COVID-19.

## Conclusions and discussion

The ongoing COVID-19 pandemic poses new problems to robustness studies on complex supply network systems. Supply chain viability—a new research stream—has been attracting more attention from supply chain management scholars. It is not about a company or a single supply chain but the entire supply system to survive the disruptions of a global pandemic. The strategies of mitigating and recovering from disruptions will be different from those of coping with traditionally natural disasters and political crises. The present study empirically investigates one Warp Knitting Industrial Zone of China and obtains two meaningful findings from its revenue growth data in 2020 after COVID-19. The more productive hub manufacturers will be impacted more severely and negatively than non-hub ones, which is conceptualized as the “*Hub Paradox*.” The other is that hub manufacturers have more comprehensive cooperation with non-hub manufacturers than classical supply chain management theories have suggested.

Inspired by Ivanov’s SCV theory, an underload cascading failure model is adapted to investigate supply chain viability and adaptation against disruptions caused by COVID-19. In summary, the results of numerical simulations demonstrate that when the load decreases beyond a certain threshold, the viability will drop critically, which is known as the phenomenon of phase transition, in both ER and BA supply networks. Besides, the supply chain viability depends on two aspects. One is the adaptive capability of manufacturers themselves, and the other is the adaptive capability of the connections of the supply network. Referring to Sects. 4.1, 4.3, and 4.4, the *capacity adjustment parameter* is the most important factor to improve the situation of hub viability. It is associated with the adaptive capability of manufacturers themselves. The larger the *capacity adjustment parameter* is, the stronger the adaptive capability of the manufacturers. Increasing the adaptive capability of manufacturers will leverage the hub viability regardless of the restrictions of the *upper bound factor* and the *lower bound factor*. In other words, it is the adaptive capability rather than the capability interval that plays the crucial role in improving the supply chain viability. On the other hand, referring to Sect. 4.2, the larger redundant capability of non-hubs will facilitate hubs to adopt more loads from them to cover the losses of underload cascading failures. However, this scenario will happen only when hubs and non-hubs have comprehensive cooperation relations. It is associated with the adaptive capability of the connections of the supply network. The more alternative connections there are, the more likely supply chain viability is to be leveraged. In other words, the supply network prefers to an intertwined structure to survive disruptions. Finally, referring to Sect. 4.5, the comparison of two models demonstrates that enhancing cooperative relations between hub and non-hub manufacturers will leverage the entire supply network viability, especially for BA networks.

Efficiency and robustness are two sides of the same coin in supply chain management. Large international companies prefer to cooperate with large manufacturers to minimize procurement costs. On the other hand, to deal with possible interruptions, they have to establish resilient supply chains by increasing redundant resources. However, these classical strategies are invalid for COVID-19. The disruptions caused by COVID-19 are different from previous disasters. COVID-19 leads to an overall demand decline, which has a greater impact on larger international companies. When larger companies shrink their orders, they will lose the scale advantage. Subsequently, closely connected hub manufacturers will be directly affected. If the hub manufacturers only depend on larger companies, they will suffer great losses and even go bankrupt. The “*Hub Paradox*” occurs.

On the other hand, real-world supply networks are mostly complex adaptive systems. There are comprehensive and complicated connections among hub manufacturers and non-hub manufacturers. Surviving COVID-19 has inspired scholars to think about supply chain resilience more dynamically and systematically than ever. Therefore, the findings of the present study provide two implications. To improve supply chain viability, manufacturers need to establish broad connections with each other, which will provide alternative routines at a lower cost. The other is that we need to protect hub manufacturers from failing. Otherwise, hub manufacturers will be removed from the system if the load decline exceeds a certain threshold, lowering the performance of the entire supply system. Finally, we suggest that a viable supply chain design needs to consider the flexible exchange of production capacities among manufacturers. Then, the supply system can re-establish overall scale advantages instead of the hub retailers and hub manufacturers.

## Supplementary Material

Supplemental material to this paper is the dataset of “revenue growth of 249 companies in WKIZ.csv”

## Supplementary Information

Below is the link to the electronic supplementary material.

Supplementary file 1 (csv 9 KB)

## Data Availability

All data generated or analyzed during this study are included in this published article (and its supplementary files).
